# Supplementary material for: Integration in oncogenes plays only a minor role in determining the in vivo distribution of HIV integration sites before or during suppressive antiretroviral therapy
Source: PLoS Pathog. 2021 Apr 7;17(4):e1009141. doi: 10.1371/journal.ppat.1009141 (PMC8055010; doi:10.1371/journal.ppat.1009141)
Supplement: S1 Table — (PDF) [file ppat.1009141.s001.pdf]

Supplemental Tables

Table S1. Sources of Donor IS Data.

			Pre-ART			On-ART		
Study	Description (Time since infection)	Donors	All Sites	Unique sites	Amp Ratio	All Sites	Unique Sites	Amp Ratio
Coffin et al. [a]	Early ART (1-6 Mo) Adult	10	4,796	4,755	1.01	2,257	2,073	1.09
Bale et al [b]	Early ART (1.8-17 Mo) Perinatal	11	10,886	8,394	1.30	3,276	2,453	1.34
Hughes et al [c]	Late ART >3 Years) Adult	4	0	0		44,733	25,956	1.72
Maldarelli et al [d]	Late ART (>3 Years) Adult	7	0	0		2,353	1,794	1.31
McManus et al [e]	Late ART (>3 Years) Adult	4	139	134	1.04	1,326	1,060	1.25
Total		36	15,821	13,293	1.18	53,945	33,336	1.62

[a] [18]

[b] Unpublished; donors described in [33]

[c] Unpublished; donors described in [27]

[d] [21]

[e] [20]

[f] Two of these participants were also included in the on-ART data of [21]. IS in the overlapping samples were only counted once.
