# Supplementary material for: Integration in oncogenes plays only a minor role in determining the in vivo distribution of HIV integration sites before or during suppressive antiretroviral therapy
Source: PLoS Pathog. 2021 Apr 7;17(4):e1009141. doi: 10.1371/journal.ppat.1009141 (PMC8055010; doi:10.1371/journal.ppat.1009141)
Supplement: S2 Table — (PDF) [file ppat.1009141.s002.pdf]

Table S2. Distribution of IS as a function of gene expression (TPM) [a]

Target Genes		PBMC			Pre ART			On ART		
TPM	% of genome	% of Sites	Sites/Mb	Bias [b] (p)	% of Sites	Sites/Mb	Bias (p)	% of Sites	Sites/Mb	Bias [b] (p)
Not in Gene	62.0%	15.9%	32		20.4%	1.4		22.8%	3.94	
<0.5	15.7%	1.3%	10	-0.031 (.02)	2.5%	0.68	-0.10 (.83)	3.0%	2.03	-0.037 (013)
1-10	9.9%	9.9%	125	0.0028 (.51)	10.3%	4.5	-0.016 (.39)	11.8%	12.77	-0.066 (4.2x 10^{-5})
10.5-30	6.8%	26.4%	482	0.0007 (.42)	24.6%	15.4	-0.073 (.02)	24.0%	37.57	-0.097 (6.4x 10^{-18})
>30	5.7%	46.6%	1,030	0.0017 (.23)	42.2%	31.9	-0.061 (.01)	38.5%	72.89	-0.13 (4.9x 10^{-47})
Total	100%	100.0%	125	0.0012 (.24)	100.0%	4.3	-0.060 (.002)	100.0%	10.71	-0.10 (1.7x 10^{-63}
In genes	38.0%	84.1%	276	0.0011 (.28)	79.6%	9.0	0.0011 (.0015)	77.3%	21.7	-0.105 (1.8 x 10^{-63})
In expressed genes	22.3%	82.8%	463	0.0015 (.19)	77.1%	14.8	0.0015 (.0021)	74.3%	35.6	-0.108 (1.8 x 10^{-63})

[a] Partial Data for Figure 1.

[b] (With the Gene – Opposite to the Gene)/(With the Gene + Opposite to the Gene).

P (Binomial) in parentheses.
