# Supplementary material for: Integration in oncogenes plays only a minor role in determining the in vivo distribution of HIV integration sites before or during suppressive antiretroviral therapy
Source: PLoS Pathog. 2021 Apr 7;17(4):e1009141. doi: 10.1371/journal.ppat.1009141 (PMC8055010; doi:10.1371/journal.ppat.1009141)
Supplement: S3 Table — (PDF) [file ppat.1009141.s003.pdf]

**Table S3. Genes in which proviruses oriented opposite to the host gene were strongly selected in the on-ART dataset**

| Gene | With | Against | Orientation p value [a] | Total in PBMC | Relative to PBMC | Relative to PBMC [b] p value | combined p value |
|---|---|---|---|---|---|---|---|
| *HNRNPM* | 1 | 12 | $1.7 \times 10^{-3}$ | 549 | 0.33 | $1.8 \times 10^{-7}$ | $3.1 \times 10^{-10}$ |
| *SMG6* | 6 | 22 | $1.9 \times 10^{-3}$ | 701 | 0.56 | $9.0 \times 10^{-5}$ | $1.7 \times 10^{-7}$ |
| *SLC6 A16* | 8 | 19 | $2.6 \times 10^{-2}$ | 112 | 3.37 | $6.3 \times 10^{-6}$ | $1.6 \times 10^{-7}$ |
| *ATF7IP* | 1 | 22 | $2.9 \times 10^{-6}$ | 196 | 1.64 | $9.1 \times 10^{-2}$ | $2.6 \times 10^{-7}$ |
| *VMP1* | 10 | 21 | $3.5 \times 10^{-2}$ | 751 | 0.58 | $9.1 \times 10^{-5}$ | $3.2 \times 10^{-6}$ |
| *NPLOC4* | 14 | 22 | $1.2 \times 10^{-1}$ | 826 | 0.61 | $4.5 \times 10^{-4}$ | $5.4 \times 10^{-5}$ |

[a] p values are binomial, not corrected for multiple comparisons.

[b] Corrected for the relative total number of unique sites
