# Supplementary material for: Integration in oncogenes plays only a minor role in determining the in vivo distribution of HIV integration sites before or during suppressive antiretroviral therapy
Source: PLoS Pathog. 2021 Apr 7;17(4):e1009141. doi: 10.1371/journal.ppat.1009141 (PMC8055010; doi:10.1371/journal.ppat.1009141)
Supplement: S4 Table — (PDF) [file ppat.1009141.s004.pdf]

Table S4. **Genes with the largest clusters in 10 kb windows in on-ART samples** [a]

Number	Gene	Shown in	chr	With Gene	Against Gene	Total	Binomial p	Total in PBMC	Normalized Ratio On Art/PBMC
1	*ASH1L*	Fig S3A	1	9	5	14	0.12	149	1.3
2	*FKBP5*	Fig S3B	6	22	27	49	0.28	762	0.9
3 [b]	***BACH2***	**Fig S3C**	**6**	**71**	**20**	**91**	**3.6 x 10^{-8}**	**132**	**9.6**
4	*FNBP1*	Fig S3D	9	24	34	58	0.12	624	1.3
5	*NEAT1*	Fig S3E	11	23	18	41	0.27	393	1.5
6	*KDM2A*	Fig S3F	11	44	46	90	0.46	1530	0.8
7	***MKL2***	**Fig S3G**	**16**	**34**	**1**	**35**	**1.0 x 10^{-9}**	**11**	**44**
8	*ITGAL*	Fig S3H	16	4	16	20	0.0059	232	1.2
9	*NAA38*	-	17	17	18	35	0.50	453	1.1
10	*IKZF3*	-	17	28	26	54	0.45	1077	0.7
11	***STAT5B***	**Fig S1**	**17**	**197**	**71**	**268**	**3.5 x 10^{-15}**	**562**	**6.7**
12	*RNF157*	-	17	26	20	46	0.23	668	1.0
13	*TNRC6C*	-	17	20	29	49	0.13	687	1.0
14	*ABCA7*	-	19	6	5	11	0.50	77	2.0
15	*POLR2E*	-	19	0	3	3	0.13	105	0.40
16	*DNMT1*	-	19	14	17	31	0.36	445	1.0
17	*CEACAM21*	-	19	12	19	31	0.14	327	1.3
18	*CARD8*	-	19	12	26	38	0.017	646	0.8
19	***MKL1***	**Fig 4C**	**22**	**53**	**30**	**83**	**0.076**	**331**	**3.5**

[a] Results from an unbiased search of the on-ART data for the most IS in a sliding 10-kb window. Genes in which HIV integration provides a selective advantage are shown in **boldface**.

[b] Two adjacent clusters.
