# Supplementary material for: Integration in oncogenes plays only a minor role in determining the in vivo distribution of HIV integration sites before or during suppressive antiretroviral therapy
Source: PLoS Pathog. 2021 Apr 7;17(4):e1009141. doi: 10.1371/journal.ppat.1009141 (PMC8055010; doi:10.1371/journal.ppat.1009141)
Supplement: S1 Fig — (PDF) [file ppat.1009141.s005.pdf]

Supplemental Figures

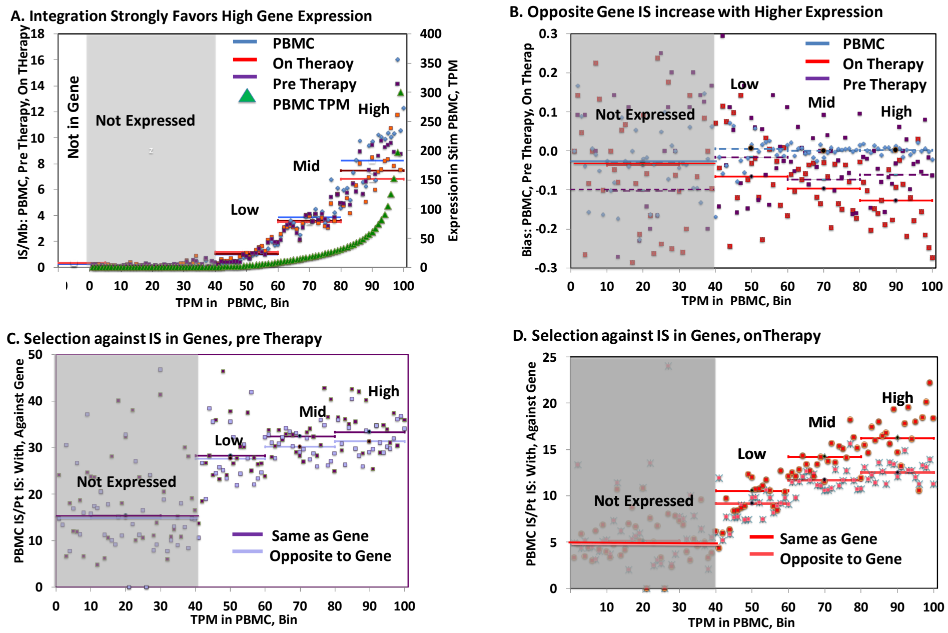

Figure S1. Gene expression and integration. This figure shows the same analysis as Figure 1, with the 100-bin IS analysis included. The complete set of ca 22,000 RefSeq genes was modified to remove overlaps (see Materials and Methods). The non-overlapping genes were divided into 100 bins, or 4 bins, based on the RNA-seq analysis (TPM) of the in vitro infected PBMC. The 100-bin data are shown in green triangles panel A. The combined IS data are shown for the genes in each of the 4 bins in all panels for PBMC (blue), pre-ART donors (plum) and on-ART donors (red). Darker colors in C and D indicate proviruses oriented in the same direction as the host gene; lighter colors are in the opposite orientation. **A**. Total IS density (sites/Mb) in each bin, normalized to the average for the whole genome (125 sites/Mb for PBMC, 4.28 sites/Mb pre-ART, and 10.7 sites/Mb on-ART). **B**. The orientation bias for the proviruses was calculated for each bin as (proviruses with gene-proviruses opposite to the gene)/(total proviruses). Dashed lines indicate p values (binomial) >0.05. **C** and **D**. Ratios of proviruses per bin for the pre-ART (**C**) or on-ART (**D**) samples. Note that the higher the ratio, the smaller the number of sites in the donor samples relative to the in vitro infected PBMC samples.
