# Supplementary material for: Integration in oncogenes plays only a minor role in determining the in vivo distribution of HIV integration sites before or during suppressive antiretroviral therapy
Source: PLoS Pathog. 2021 Apr 7;17(4):e1009141. doi: 10.1371/journal.ppat.1009141 (PMC8055010; doi:10.1371/journal.ppat.1009141)
Supplement: S2 Fig — (PDF) [file ppat.1009141.s006.pdf]

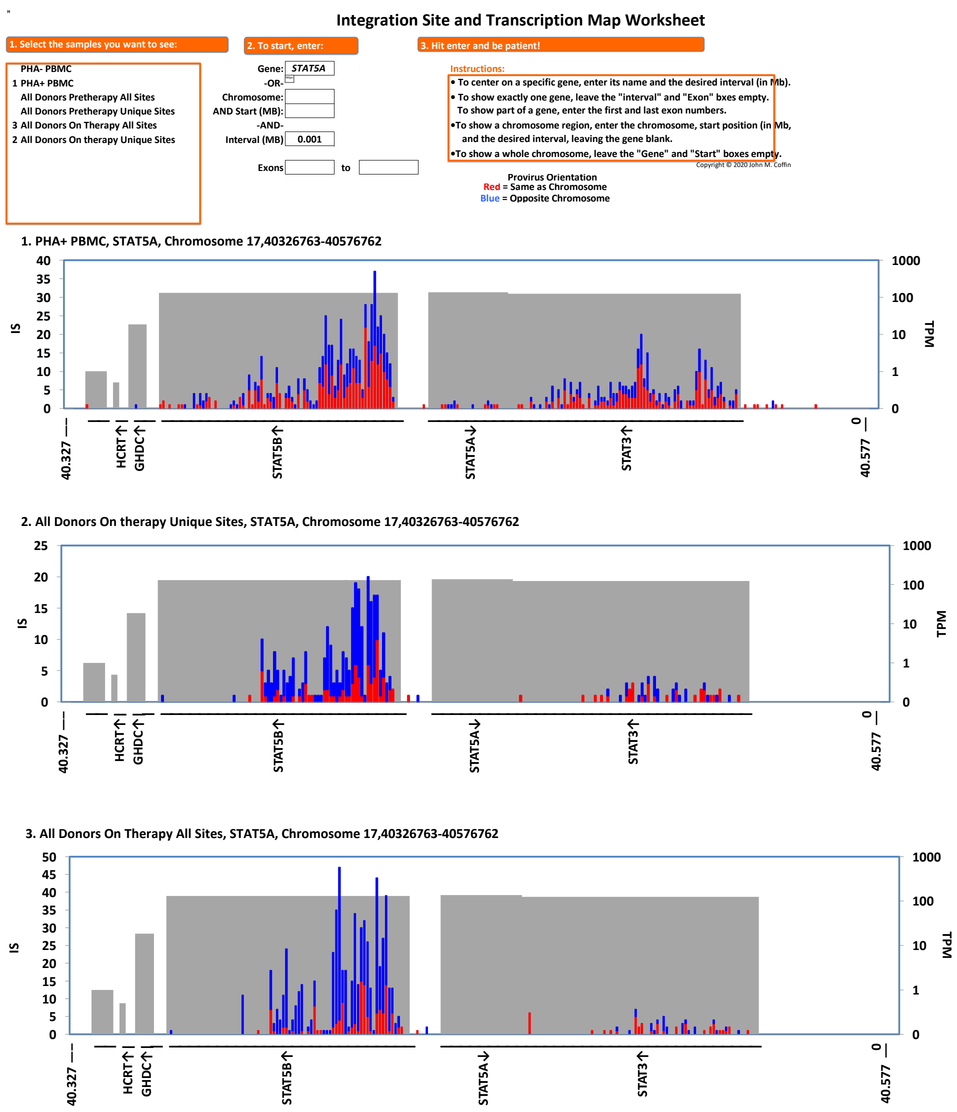

Figure S2. Application for visualization of IS and expression data. The Figure shows a screenshot of the Microsoft Excel-based application we developed to display IS data at all scales from a whole chromosome down to as few as 250 bp. The box (1) at upper left contains a list of all available datasets (either used in this study or added by the user), which are on an attached worksheet. Datasets to be displayed are selected by entering a number 1-5 before the name. As described in the instructions (3) at the upper right, there are several display options available using the entry boxes (2). To show a selected region of a chromosome, the user leaves the gene name blank and enters the chromosome, starting position (in Mb), and bin size (in Mb). To show a region centered on a single gene (as in this example), the user enters the gene name in the top box, and the bin size in the bottom box. (The middle two boxes are ignored). To show a whole chromosome, the user leaves the first and third boxes empty. Pressing enter and clicking on an empty box then starts the analysis, which takes about 15 seconds per dataset on a 5-year-old MacBook Pro running OS 10.13.6 and Excel 2011. The plots show the selected region divided into 250 bins of the chosen size, with genes from the RefSeq dataset (slightly modified by removing overlaps) shown on the X-axis, with their transcriptional orientation relative to the chromosome numbering indicated by the arrows above the names, and lines indicating their extent. The gray boxes show the expression for each gene, as TPM (right Y-axis). The stacked colored bars show the number of proviruses in each bin, with those whose orientation matches the numbering of the chromosome in red, and those in the opposite orientation in blue. Each plot is labeled with the name of the dataset, the chromosome number, and range of sequence displayed.

The application runs on both MacOS and Windows computers. It consists of 3 worksheets: the display sheet shown here, the gene list with TPM data used, a second gene list with exon and TPM data, and the is data used in this paper. Additional datasets can be easily added by the user and will be automatically incorporated into the list in box 1.The application is available as Supplemental File 1.
