# Supplementary material for: Integration in oncogenes plays only a minor role in determining the in vivo distribution of HIV integration sites before or during suppressive antiretroviral therapy
Source: PLoS Pathog. 2021 Apr 7;17(4):e1009141. doi: 10.1371/journal.ppat.1009141 (PMC8055010; doi:10.1371/journal.ppat.1009141)
Supplement: S4 Fig — (PDF) [file ppat.1009141.s008.pdf]

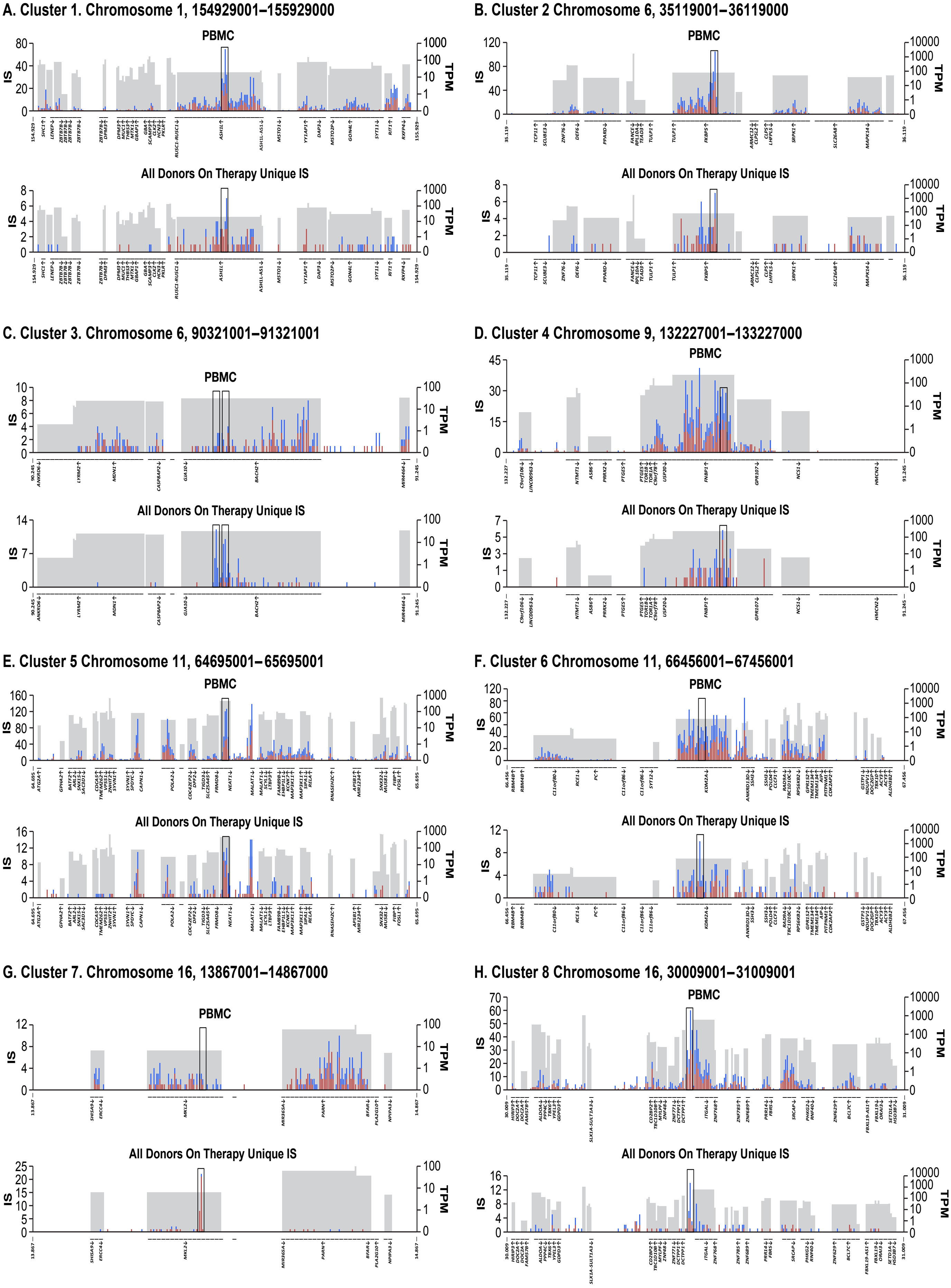
A. Cluster 1. Chromosome 1, 154929001–155929000
B. Cluster 2 Chromosome 6, 35119001–36119000
C. Cluster 3. Chromosome 6, 90321001–91321001
D. Cluster 4 Chromosome 9, 132227001–133227000
E. Cluster 5 Chromosome 11, 64695001–65695001
F. Cluster 6 Chromosome 11, 66456001–67456001
G. Cluster 7. Chromosome 16, 13867001–14867000
H. Cluster 8 Chromosome 16, 30009001–31009001
PBMC
All Donors On Therapy Unique IS
IS
TPM

F**igure S4. Clusters of IS not associated with cell growth or survival.** The largest clusters of IS were identified by using an unbiased procedure in which each chromosome in the on-ART IS dataset was scanned with a sliding window to find the regions with the largest number of proviruses in any 10-kb stretch. Of the he top hits listed in Table S3, eight are shown here, with the cluster found in the on-ART data indicated by the box, Each of the maps shows a 1-Mb region (4 kb/bin) around the cluster. **A.** Chromosome 1, unannotated region between *ASH1L* and *ASH1l-AS1*. **B.** *FKBP5*. **C**. *BACH2* (Note that s2 clusters were found). ***D.*** *FNBP1*. **E.** *NEAT1* (Note also the cluster in the nearby *MALAT*1). **F.** *KDM2A*. **G**. *MKL2*. **H.** Unannotated region between *DCTPP2* and *ITGAL*.
