# Supplementary material for: Integration in oncogenes plays only a minor role in determining the in vivo distribution of HIV integration sites before or during suppressive antiretroviral therapy
Source: PLoS Pathog. 2021 Apr 7;17(4):e1009141. doi: 10.1371/journal.ppat.1009141 (PMC8055010; doi:10.1371/journal.ppat.1009141)
Supplement: S5 Fig — (PDF) [file ppat.1009141.s009.pdf]

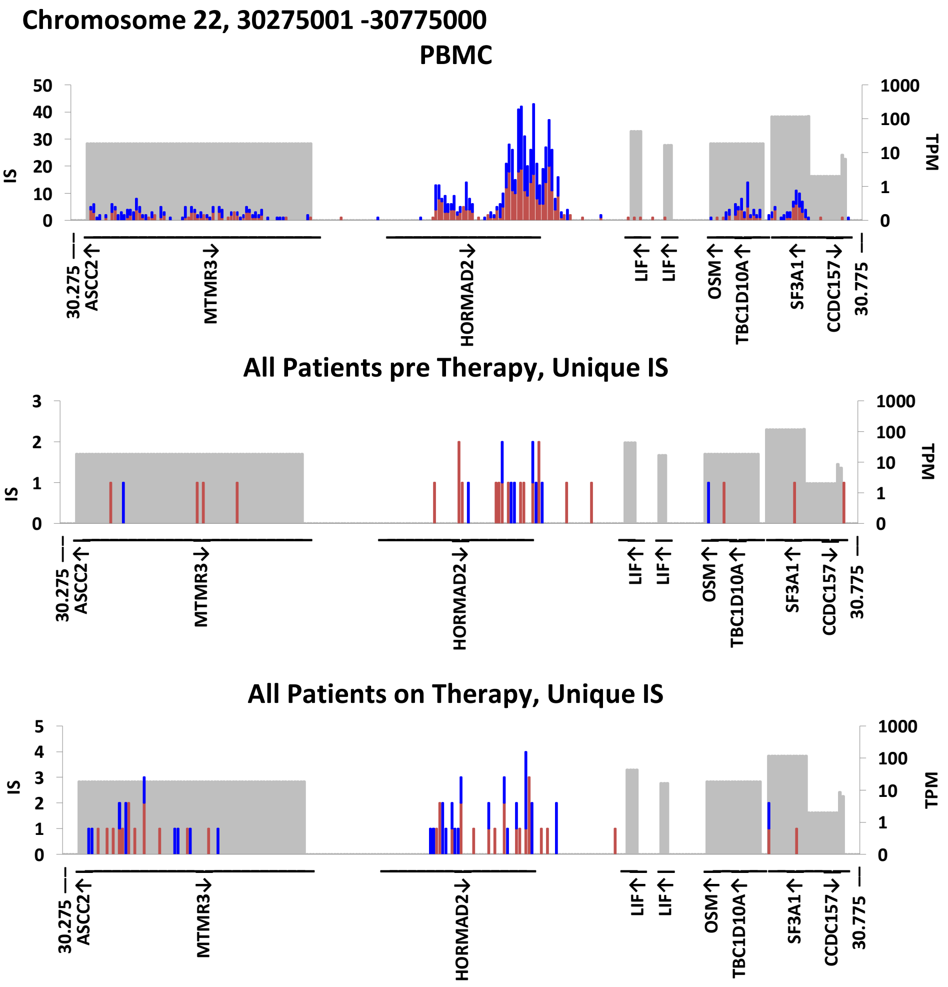

Figure S5. Integration into a non-expressed gene. The figure shows IS in *HORMAD2,* a gene involved in meiosis, which is not detectably expressed in PBMC or a number of other cell types in vitro, but is one of the few non-expressed genes that are good targets for HIV-1 integration.
