# Supplementary material for: Integration in oncogenes plays only a minor role in determining the in vivo distribution of HIV integration sites before or during suppressive antiretroviral therapy
Source: PLoS Pathog. 2021 Apr 7;17(4):e1009141. doi: 10.1371/journal.ppat.1009141 (PMC8055010; doi:10.1371/journal.ppat.1009141)
Supplement: S6 Fig — (PDF) [file ppat.1009141.s010.pdf]

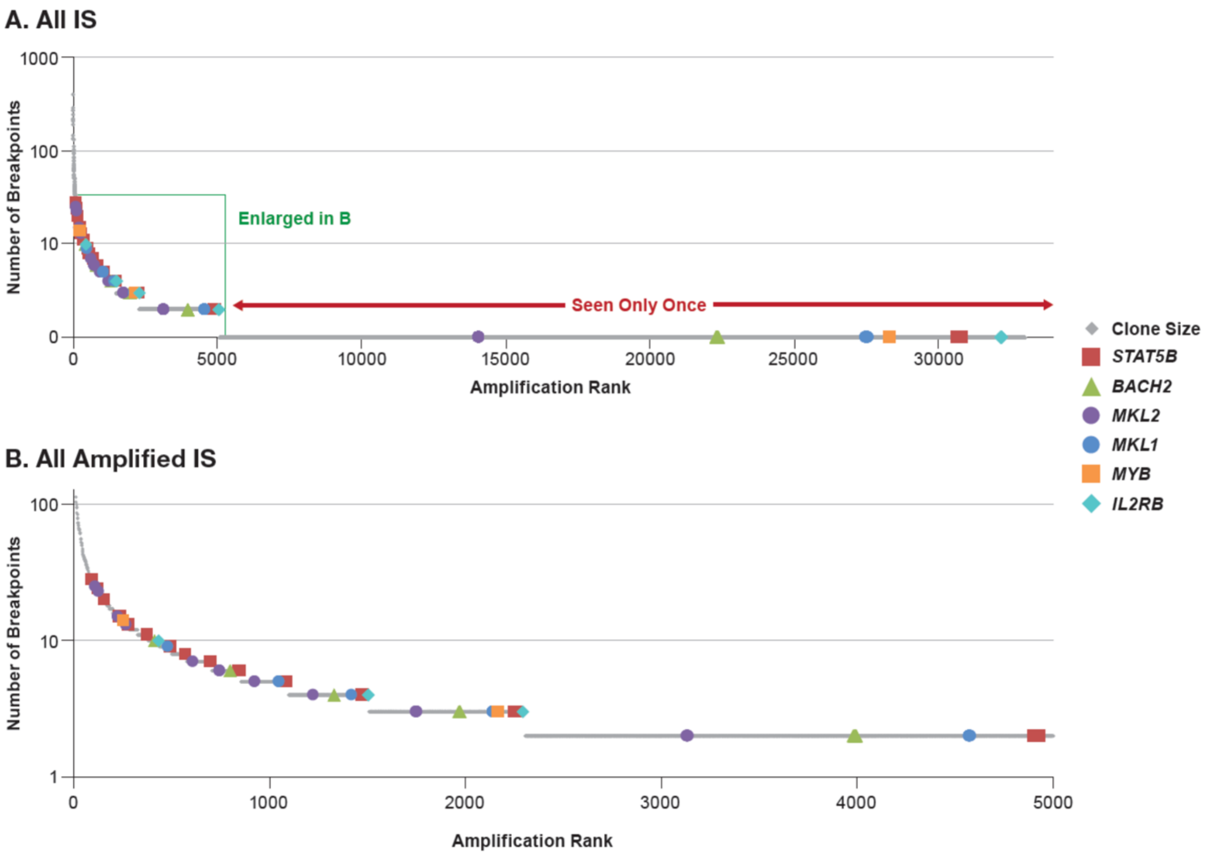

Figure S6. IS Ranked by Clonal Amplification. A. All 33,000 on-ART IS are plotted as a function of the number of breakpoints (i.e., the amplification ratio) per site. IS in the 6 selective genes are indicated by the various symbols as shown in the key. The dashed line indicates the region enlarged in **B.**
