# Supplementary material for: Integration in oncogenes plays only a minor role in determining the in vivo distribution of HIV integration sites before or during suppressive antiretroviral therapy
Source: PLoS Pathog. 2021 Apr 7;17(4):e1009141. doi: 10.1371/journal.ppat.1009141 (PMC8055010; doi:10.1371/journal.ppat.1009141)
Supplement: S7 Fig — (PDF) [file ppat.1009141.s011.pdf]

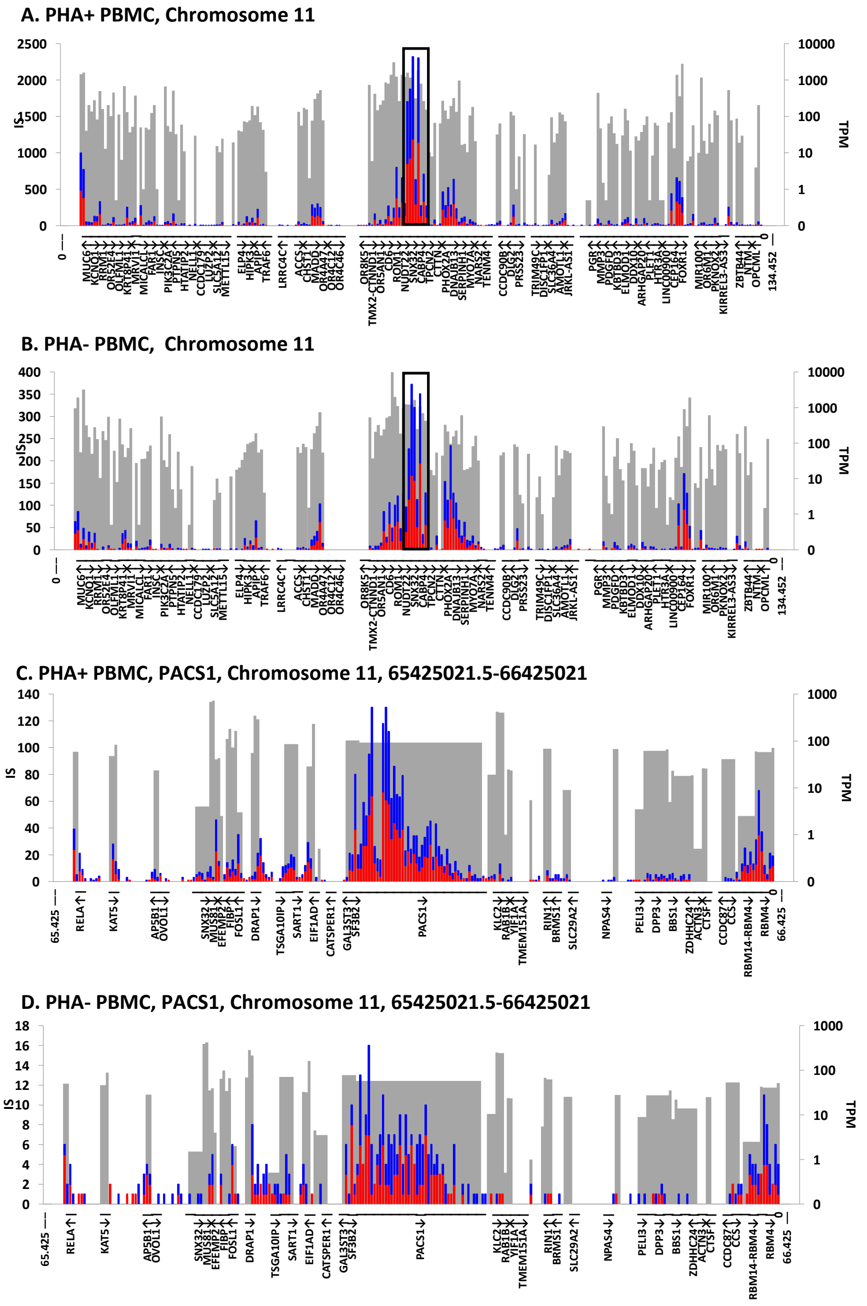


**Figure S7. Effect of PHA stimulation on transcription and integration.** Donor PBMC were infected with HIV as in the other figures in the presence (**A, C**) or absence (**B, C**) of PHA, as described in Materials and Methods, subjected to ISA, and analyzed as in other figures. Panels **A** and **B** show the distribution of gene expression (grey) and IS over the whole 134 Mb chromosome 11, and **C** and **D** a 1-Mb strongly targeted region indicated by the boxes .in **A** and **B**. Note that the distribution of transcripts and IS are virtually indistinguishable between the two conditions except that PHA stimulation increased the number of IS by about 8-fold.
