# Supplementary material for: Integration in oncogenes plays only a minor role in determining the in vivo distribution of HIV integration sites before or during suppressive antiretroviral therapy
Source: PLoS Pathog. 2021 Apr 7;17(4):e1009141. doi: 10.1371/journal.ppat.1009141 (PMC8055010; doi:10.1371/journal.ppat.1009141)
Supplement: S3 Fig — (PDF) [file ppat.1009141.s007.pdf]

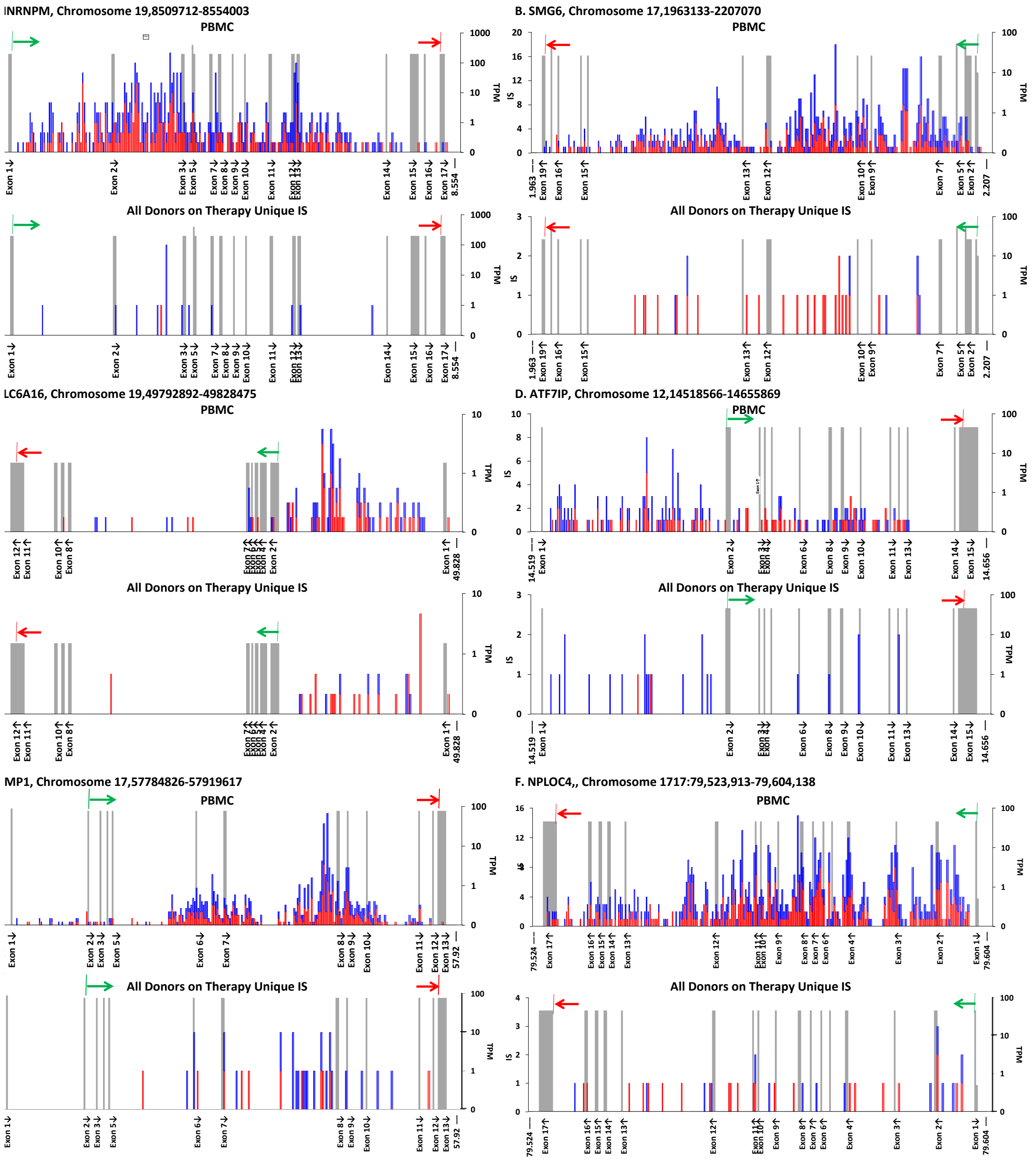

Figure S3. Genes with high frequency of proviruses integrated in the opposite transcriptional orientation. The maps show the distribution of the proviruses in the top six genes with the strongest bias for orientation against the gene in the on-ART samples (Table S3). In all panels, the top map shows the data for PBMC infect in vitro, with the data for on-ART samples beneath. Color coding is as in the other figures: gray, expression (TPM) in in vitro infected PBMC; red, proviruses/bin in the same orientation as the chromosome; blue, proviruses/bin in the opposite orientation. The orientation of the gene relative to the chromosome is shown by the arrow under the gene name at the bottom of each map. The Gene Browser plot, showing introns and exons is shown at the top of each map. **A.** *HNRNPM.* 177 bp/bin. **B** *SMG6.* 976 bp/bin. **C.** *SLC6A16.* 1.34 kb/bin. **D.** *ATF7IP***.** 549 bp/bin. **E.** *VMP1.* 539 bp/bin.*NPLOC4*.321 bp/bin
